# The IKK-binding domain of NEMO is an irregular coiled coil with a dynamic binding interface

**DOI:** 10.1038/s41598-019-39588-2

**Published:** 2019-02-27

**Authors:** Adam H. Barczewski, Michael J. Ragusa, Dale F. Mierke, Maria Pellegrini

**Affiliations:** Department of Chemistry, Dartmouth College, Hanover, NH 03755 USA

## Abstract

NEMO is an essential component in the activation of the canonical NF-κB pathway and exerts its function by recruiting the IκB kinases (IKK) to the IKK complex. Inhibition of the NEMO/IKKs interaction is an attractive therapeutic paradigm for diseases related to NF-κB mis-regulation, but a difficult endeavor because of the extensive protein-protein interface. Here we report the high-resolution structure of the unbound IKKβ-binding domain of NEMO that will greatly facilitate the design of NEMO/IKK inhibitors. The structures of unbound NEMO show a closed conformation that partially occludes the three binding hot-spots and suggest a facile transition to an open state that can accommodate ligand binding. By fusing coiled-coil adaptors to the IKKβ-binding domain of NEMO, we succeeded in creating a protein with improved solution behavior, IKKβ-binding affinity and crystallization compatibility, which will enable the structural characterization of new NEMO/inhibitor complexes.

## Introduction

The nuclear factor κB (NF-κB) transcription factor is key to the regulation of multiple cellular processes, including cell proliferation and survival, B-cell and T-cell maturation, and inflammatory response^1^. In the canonical NF-κB pathway, NF-κB dimers are sequestered in the cytoplasm by the inhibitor of κB molecules (IκB). Activation of the signaling pathway by stimuli including cytokines, pathogens, stress or ultraviolet radiation, is mediated by an essential node, the IKK complex, composed of the NF-κB essential modulator (NEMO) and the IKKα and IKKβ kinases^2^. The IKK complex phosphorylates IκB leading to ubiquitination and proteosomal degradation^3^ and allowing NF-κB to translocate to the nucleus and activate target genes.

Mis-regulated NF-κB activity has been linked to human diseases encompassing inflammatory and autoimmune diseases and cancer^4–7^ and modulation of the NF-κB pathway has therefore been the focus for possible therapeutic development^8,9^. The NF-κB pathway presents multiple possible levels of intervention for inhibition, among which targeting the NF-κB inducers TNFα, IL-1 and IL-6^10,11^, inhibition of cell surface receptors (e.g., TNFR, IL-1R)^12,13^, inhibition of IKKβ, inhibition of IκBα degradation^14,15^ or further downstream inhibition of NF-κB nuclear translocation or DNA binding^16^. Inhibition of the protein-protein interaction between NEMO and IKKβ represents an attractive alternative strategy due to the crucial role of NEMO and its selective involvement in the canonical NF-κB pathway.

NEMO^17^ is a 419 amino acid protein containing two coiled-coil domains, a leucine zipper domain, and a zinc finger domain in an elongated dimeric structure^18^. The minimal binding domain necessary to recognize IKKβ was identified as residues 44–111 and the structure was reported in complex with the NEMO-binding domain of IKKβ (residues 701–745)^19^. The structure displays a four helical bundle in which the two helices of the NEMO (44–111) dimer are intercalated by the two helices of IKKβ with an extensive interaction interface. Analysis of this structure coupled with Ala-scanning mutagenesis identified three hot-spot regions for binding within IKKβ, with the strongest interaction occurring at the very C-terminus of IKKβ (residues 734–742)^20^.

The structure provides detailed insight into the earlier discovery of a small peptide inhibitor of the NEMO/IKKβ interaction, named the “NEMO binding domain” or NBD peptide and corresponding to the IKKβ sequence 737–742^21^. Despite the weak affinity for NEMO, the peptide has proven to be an important physiological tool and its efficacy has been demonstrated in over 70 cellular and *in vivo* studies. An experimentally derived structure of unliganded NEMO or of NEMO in the presence of small molecule inhibitors would provide the needed structural framework for the structure-based development of improved NEMO inhibitors. The task of determining the structure of the unliganded IKKβ-binding domain of NEMO has been challenging, as the domain, when truncated from the full-length protein, is conformationally heterogenous and appears only partially folded^19,22^. Longer constructs of NEMO or full-length NEMO have proven equally difficult to handle and no structure by NMR or X-ray crystallography has been described.

We have previously reported the design and characterization of a coiled-coil stabilized NEMO construct encompassing the IKKβ-binding region fused to two ideal dimeric coiled-coil adaptors, at the N and C-terminus. The engineered NEMO achieved high stability and structural homogeneity and rescued high affinity binding for IKKβ *in vitro* and in cells^22^.

**Figure 1:**
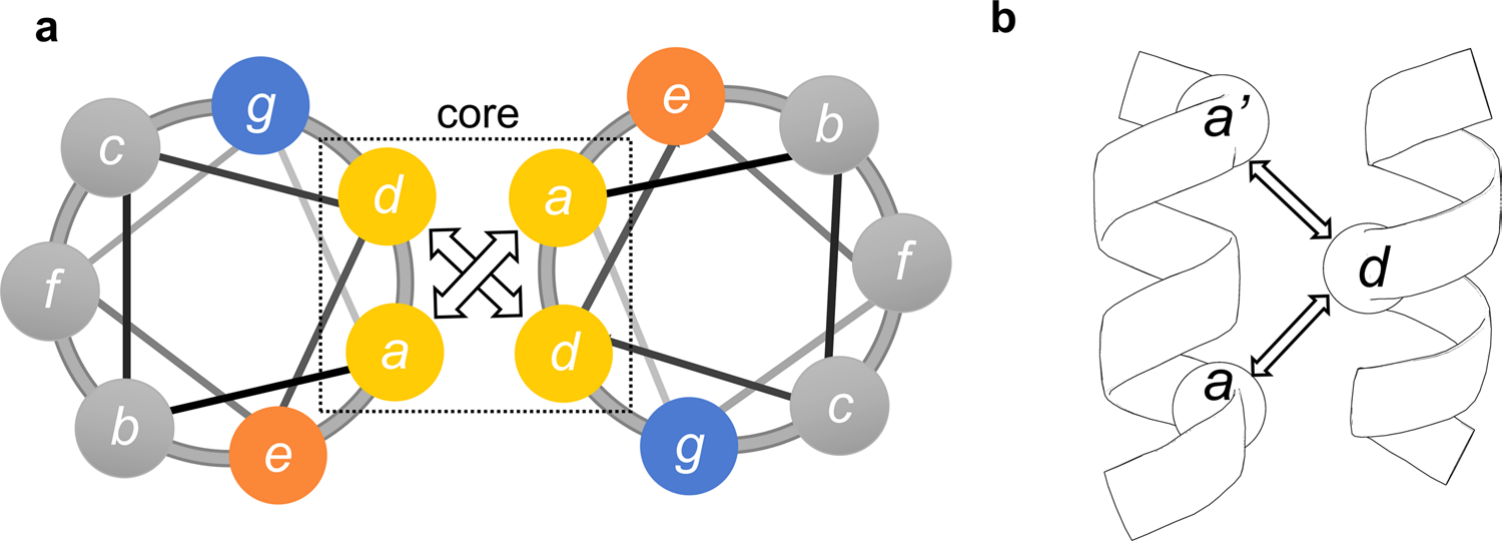
Coiled-coil heptad repeats. (**a**) *a-g* heptad repeats in a typical coiled coil. (**b**) coiled-coil interhelical distances: *da*, *da’* (Cα are depicted as sphere, *a’* is the start of the next heptad).

The coiled coil is a common structural motif and consists of, in this case, two helices wrapped around each other to form a supercoil^23^. Each helix is characterized by periodic heptad repeats, usually denoted (*a-b-c-d-e-f-g*) in one helix and (*a’-b’-c’-d’-e’-f’-g’*) in the other (Fig. 1a). Residues *a* and *d* are typically non-polar amino acids buried at the interface between the two helices, while *e* and *g* are charged amino acids which contribute to the dimeric coil stability through salt bridges^24^. In our design the heptad repeats of a GCN4-based ideal coiled coil^25^ were matched to the predicted heptads of the NEMO sequence 51–112, to create a continuous and seamless coiled coil. The desired outcome was to increase the crystallization potential of the NEMO construct in two ways: by increasing the intrinsic stability (or decreasing conformational heterogeneity) and possibly by facilitating crystallization through the GCN4 adaptors portion of the protein. Coiled-coil adaptors have been similarly utilized to both increase stability, improve solution behavior and facilitate crystallization for trimeric coiled coils and antibody fragments^26,27^.

Here we report the structure of the unliganded N-terminal domain of NEMO, utilizing the coiled-coil stabilized NEMO construct modified by four additional point mutations, designed to improve solution behavior and crystal packing. The crystal structure reveals that NEMO folds in an irregular dimeric coiled coil in the absence of IKKβ, with discontinuities in the heptad repeats causing an underwinding of the supercoil in the ligand binding region. The structure opens new possibilities for the design and optimization of inhibitors that target the NEMO binding pockets, particularly small molecules or peptides that exploit the observed conformational flexibility in this region.

## Results

### NEMO Construct Design and Preparation

We started from our previously reported construct of the minimal IKKβ binding domain of NEMO, NEMO (44–111), fused to a homodimeric coiled-coil adaptor sequence based on GCN4 at both the N- and C-terminus^22^. In this study the construct was further improved by adding the following mutations (listed in Supplementary Table S1). All constructs incorporate sequence 51–112 of NEMO. Non-essential cysteines^28^ were mutated (C76A and C95S) to prevent disulfide induced oligomerization (NEMO-CC). Mutations E56A and E57A, suggested by the Surface Entropy Reduction Server SERp^29^, were chosen to promote crystal lattice contacts between NEMO dimers, and as unlikely to affect IKKβ binding affinity (NEMO-EEAA). The mutation I65M was introduced to provide an additional site for selenomethionine labeling, to aid in phase determination (NEMO-I65M). Ile65 was chosen for its expected buried position within the coiled coil, providing a more rigid methionine than that of the natural sequence Met94.

We expressed in *E. coli* and purified to homogeneity all NEMO and IKKβ constructs to conduct biochemical and structural studies. The mutant proteins show more stable dimer formation than the wild type NEMO (44–111), as observed by bands at the dimer molecular weight in SDS-PAGE gels^22^. The NEMO constructs have short retention times on size exclusion chromatography (SEC) due to their elongated structure, and all elute at an apparent molecular weight of 44 kDa^19,22^.

### The coiled-coil NEMO constructs are stable and bind IKKβ with high affinity

**Figure 2:**
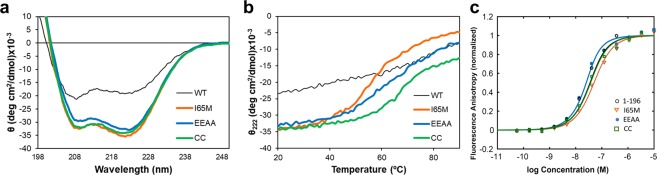
The coiled-coil NEMO constructs are stable and bind IKKβ with high affinity. (**a**) Overlay of the CD spectra for the NEMO constructs, showing the high coiled-coil content. WT NEMO(44–111) in black. (**b**) Temperature unfolding curves as monitored by CD at 222 nm. (**c**) Binding affinity of NEMO constructs for FITC-IKKβ_KKRR_(701–745) by fluorescence anisotropy; lines represent the curve fitting. GST-NEMO(1–196) in black.

We assessed the effect of the mutations of the three NEMO constructs on secondary structure by circular dichroism (CD). The NEMO-CC, NEMO-EEAA and NEMO-I65M constructs are characterized by a calculated helical content of 85%, 82% and 87%, resulting in helix stabilization compared to the unmodified NEMO (44–111) construct (54% helix)^22^. The spectra of all three constructs indicate coiled-coil character, as measured by a ratio of the ellipticity at 222 and 208 nm larger than 1 (Fig. 2a). NEMO-EEAA and NEMO-I65M display high stability to thermal denaturation, with a sigmoidal transition characteristic of cooperative unfolding and melting temperatures of 69 and 71 °C, while the I65M mutant melts cooperatively at 54 °C (Fig. 2b). The unmodified NEMO (44–111) construct displays instead a shallow melting curve, indicative of the progressive loss of α-helical secondary structure and consistent with its only partially folded and conformationally heterogeneous nature^30^.

The binding affinity of the mutants for IKKβ, was measured by fluorescence anisotropy, using recombinant, FITC labelled IKKβ_KKRR_(701–745). FA analysis provides a K_D_ = 22 ± 8 nM for NEMO-CC, K_D_ = 10 ± 8 nM for NEMO-EEAA and a K_D_ = 35 ± 11 nM for NEMO-I65M (Fig. 2c). These values are similar to what we measured for the reference construct GST-NEMO (1–196), with a K_D_ = 12 ± 4 nM and comparable to what reported for full length NEMO with a K_D_ = 3.6 nM^20^. For comparison, the original NEMO (44–111) truncated construct displays a binding affinity estimated around 10 µM^22^, as a complete binding curve could not be obtained at the concentrations explored.

### Crystallization of NEMO-EEAA and NEMO-I65M

The crystal structures of NEMO-EEAA and NEMO-I65M were determined in the P 1 2_1_ 1 space group at resolutions of 1.9 Å (data anisotropically truncated to 1.88 Å, 2.10 Å and 2.55 Å along the a*, b* and c∗ axis) and 2.5 Å, respectively (Table 1). The SeMet labeled I65M mutant was prepared to facilitate the phasing of the EEAA structure, but displayed weak anomalous signal, and the structures were instead solved by molecular replacement. Composite omit maps where calculated with PHENIX for NEMO-EEAA and NEMO-I65M, excluding 10% of the atoms at a time, and compared with the final structure models, to confirm that the structures were not biased by the atomic models (Supplementary Fig. S1). The crystallographic models are complete except for the first and last residues in chain A of NEMO-EEAA, the first residue in chain A and the last residue in chain B of NEMO-I65M, for which no electron density was observed.

**Table 1:** Crystallographic data collection and refinement statistics.

	NEMO-EEAA	NEMO-I65M
**Data Collection**		
Space Group	P 1 2_1_ 1	P 1 2_1_ 1
Cell Dimensions		
*a*, *b*, *c* (Å)	64.48, 33.62, 76.87	64.41, 34.12, 77.42
*α*, *β*, *γ* (°)	90.00, 115.04, 90.00	90.00, 113.89, 90.00
Multiplicity	3.3 (3.4)*	6.5 (6.0)
Elliptical Completeness %	88.00 (54.50)**	98.43 (97.42)
I/σ(I)	5.71 (0.24)	11.64 (2.38)
R_meas_	0.10 (6.49)	0.13 (0.85)
CC _½_	0.997 (0.089)	0.998 (0.875)
**Refinement**		
Resolution Range	37.60–1.78** (1.85 – 1.78)	38.29–2.50 (2.59 – 2.50)
Reflections used in refinement	19560 (431)	10823 (1058)
Reflections used for R_free_	991 (21)	542 (53)
R_work_/R_free_	0.24/0.27	0.25/0.29
No. of atoms		
Protein	2044	2044
Water	183	59
B factors (Å^2^)		
Protein	49.12	57.59
Water	47.31	51.25
R.m.s. deviations		
Bond lengths (Å)	0.012	0.004
Bond angles (°)	1.34	0.79

*Highest resolution shell is shown in parenthesis.

**The anisotropic diffraction data for NEMO-EEAA was truncated using the STARANISO server to include all valid data (reflections with I/σ(I) of 1.2) to resolutions of: 1.88 Å, 2.10 Å and 2.55 Å along the a*, b* and c* axis respectively.

### The structure of unbound NEMO is an irregular coiled coil

**Figure 3:**
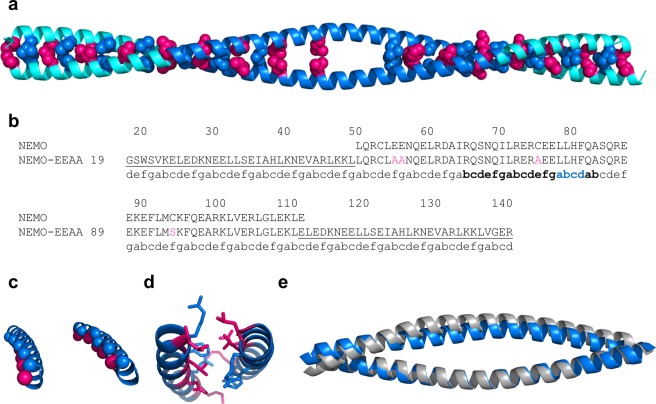
The structure of unbound NEMO is an irregular coiled coil. (**a**) The NEMO-EEAA dimer is shown as a ribbon, light blue = coiled-coil adaptors, blue = NEMO (51–112). *a* (hot pink) and *d* (blue) residues are shown in spheres. Residues in the discontinuity region (73–90) are not shown (except Leu79). (**b**) Sequence alignment of NEMO and NEMO-EEAA, the coiled-coil adaptors sequence is underlined and the mutations are highlighted in pink; the heptad repeats are marked under the sequence: the region with low coiled-coil propensity is in bold and the stutter region is in blue. (**c**,**d**) Effect of the stutter on core residue packing: the Cα of *a* and *d* residues are shown, coloring as in (**a**): (**c**) ideal coiled-coil region of NEMO (23–57), left, compared to the irregular region of NEMO (69–103), right. (**d**) the core packing is disrupted in the discontinuities region, shown the dimer of NEMO (72–92) with side chains of *a* and *d* residues in sticks (coloring as in (**a**)). (**e**) Superposition of apo NEMO-EEAA structure (blue) and NEMO (44–111) from the IKKβ-bound structure (grey, PDB: 3BRV, IKKβ is not displayed), shown as ribbons; the structures are aligned on chain A, region 44–111 only.

NEMO-EEAA is a homo-dimeric, irregular, parallel coiled coil of ∼175 Å in length. The region corresponding to the GCN4 adaptors in NEMO-EEAA is a regular coiled coil, with each heptad repeat measuring ∼1 nm along the coiled coil^31^. The regular coiled coil extends to the first two heptads of the NEMO sequence, to residue Ile65, and resumes after Phe97, encompassing the last 15 residues of the NEMO sequence and the C-terminal GCN4 adaptor. These regions are characterized by canonical hydrophobic residues in *a*-*d* positions packed as knobs-into-holes, forming the hydrophobic zipper (highlighted in Fig. 3a,b). Buried Asn residues in position *a* (Asn30, Asn58 and Asn118) create *a-a’* hydrogen bonds in this structure, imparting dimerization specificity^23,25^. Similarly, charged residues in positions *g* and *e* form stabilizing salt bridges: *g*-Glu36/*e’*-Lys41, *g*-Glu43/*e’*-Lys48, *g*-Glu124/*e’*-Lys129^23^.

The coiled-coil propensity (as predicted by PairCoils2^32^) decreases (score > 0.0025) between residues 66–103 of NEMO, corresponding to the IKKβ binding site, and the heptad repeats display discontinuities between residues 74–83 (Supplementary Table S2). The heptad definitions are used as calculated by PairCoils2 till residue 74. The irregular heptad at 72–78 and a stutter (four-residue insert, *abcd*, in blue in Fig. 3b) at 79–82 were assigned based on the experimental structure. A stutter in a coiled coil causes an underwinding of the supercoil and non-close-packed cores, resulting in increased flexibility^23^. Figure 3c,d show the stutter disrupts the alignment of *a* and *d* residues and polar residues in core positions cause non-close-packing. We observe that between residues 65–97 the structure is still a helical dimer, but the interhelical spacing ((*da* + *da’*)/2 in Fig. 1b, and Table 2) increases and the helix loses some supercoil twist. The interhelical spacing is maximum at the stutter (79–86), with a weak hydrophobic *a-a’* interaction at residues Leu79. The interhelical spacing goes from an average value of 7.6 Å in the regular coiled-coil structure to a maximum of 11.5 Å for the stutter “heptad”. As previously observed in coils discontinuous regions^25^, buried polar *a*-residues, such as lysine, can form favorable interactions with *g’* glutamate (*a*-Lys90/*g’*-Glu89, and *a*-Lys111/*g’*-Glu110) lowering the energetic penalty for burying polar residues^33^.

**Table 2:** Interhelical distances calculated from the three structures indicated. *a*,*a’* and *d* residues are as indicated in Fig. 1b.

Residue Number			NEMO-EEAA	3BRV	NEMO-I65M
a	a’	d	(da + da’)/2	(da + da’)/2	(da + da’)/2
23	30	26	7.59		7.66
30	37	33	7.84		7.71
37	44	40	7.61		7.63
44	51	47	7.68		7.53
51	58	54	7.67	7.46	7.56
58	65	61	7.64	7.96	8.00
65	72	68	7.94	9.58	9.30
72	79	75	9.61	11.84	11.60
79	86	82	11.55	12.52	12.91
83	90	86	10.11	12.34	10.47
90	97	93	8.47	10.21	8.50
97	104	100	7.16	8.27	7.43
104	111	107	7.51		7.82
111	118	114	7.82		7.82
118	125	121	7.79		7.83
125	132	128	7.58		7.72
132	139	135	7.56		7.66

In the bold area of increased interhelical distances.

### A tighter dimer for apo-NEMO-EEAA vs. IKKβ-bound NEMO

All three hot-spots for binding of IKKβ reside within, or at the boundaries of this central region of irregular coiled coil^19^. If we describe the two parallel helices of NEMO as a hot-dog bun (where the bound IKKβ would represent the actual hot-dog), the NEMO apo-structure shows a “closed bun” conformation, while the IKKβ-complex structure (PDB: 3BRV) shows an “open bun” (Fig. 3e). Notably, a second example of the “open bun” conformation is observed in the reported complex structure of NEMO with the IKKβ/α hybrid peptide (PDB: 3BRT). Despite sequence differences in the NBD and immediately preceding region of the ligand, ligand binding causes the same bun opening and a complex structure essentially identical to the NEMO/IKKβ structure^19^. Given the similarity, from now forward we will utilize the structure of the NEMO/IKKβ complex (PDB: 3BRV) for comparative analysis. We denote residues in the first and second helix of the dimer as Xxx and Xxx’ respectively. Both structures reach the point of maximum interhelical spacing at the stutter at Leu79 (see Table 2), but the IKKβ-complex structure has a more extensive opening (residues 65–97), more pronounced crescent shape, and larger interhelical spacing by 1.0 to 2.2 Å in this region. The bun opening is also reflected in the solvent exposed surface: considering the *a* and *d* residues for region 65–97, the NEMO apo structure has a solvent exposed surface of 1,249  Å^2^ and the IKKβ-bound structure of 1,863  Å^2^ (calculated removing the bound IKKβ). N-terminal of the three binding pockets (i.e., residues Leu51-Ala64) the structures of the apo and bound forms are remarkably similar, with an all atoms RMSD = 0.584 Å.

**Figure 4:**
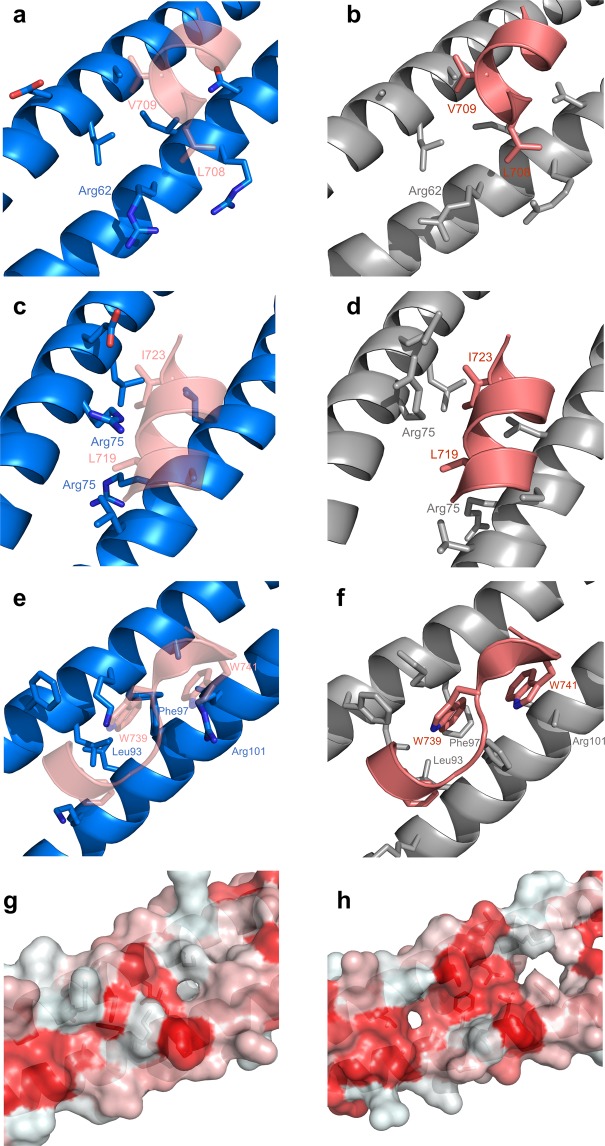
The three hot-spot residues binding pockets. NEMO-EEAA (blue, left panels), NEMO in complex with IKKβ (PDB: 3BRV; grey, right panels), only hot-spot residues of IKKβ are shown (deep salmon). The IKKβ is shown overlaid to the NEMO-EEAA structure in transparency in the left panels for reference. (**a**,**b**) Pocket I: L708 and V709 of IKKβ in sticks. NEMO’s Glu60, Leu61, Ala 64 (top helix) and Arg62′, Ile65′, Arg66′, Asn69′ (bottom helix), also in sticks. (**c**,**d**) Pocket II: L719 and I723 of IKKβ in sticks. NEMO’s Ile71, Arg75, Glu78, Leu79 (top helix) and Leu72′, Arg75′, Cys/Ala76′, Leu79′ (bottom helix), also in sticks. (**e,f**), Pocket III: F734, W739 and W741 of IKKβ in sticks, NEMO’s Phe92, Leu93, Lys96, Phe99, Ala100 (top helix) and Lys90′, Leu93′, Phe97′, Ala100′, Arg101′ (bottom helix), also in sticks; (**g**,**h**) surface rendering of pocket III of NEMO-EEAA (left) and NEMO in complex with IKKβ (IKKβ removed, right). The surface area is colored by hydrophobicity red = hydrophobic, white = polar.

The first binding hot-spot is centered on IKKβ residues L708/V709, and it involves NEMO residues Glu60, Leu61, Ala64 from chain B and Arg62′, Ile65′, Arg66′, Asn69′ from chain D in 3BRV (pocket I; IKKβ residues are denoted in a one-letter code and NEMO in a three-letter code). In this region the bound structure just starts to open up (by ∼1.6 Å at heptad 65–68). Side chain conformations are remarkably similar, with the exception of Arg62′ which moves towards the inside of the pocket. Asn69 is also shifted towards the core of the pocket, for an overall more closed conformation (Fig. 4a,b).

The second binding hot-spot (pocket II) is centered around IKKβ residues L719/I723, and is formed by NEMO residues Ile71, Arg75, Glu78, Leu79 of chain B and Leu72′, Arg75′, Cys76′, Leu79′ of chain D. Interhelical spacing shows here the greatest difference, with the apo structure closing the hot-dog bun by 2.2 Å. Side chains of NEMO pocket-lining residues assume very similar orientation to the bound structure, but the entire helices experience a shift towards the center, with the maximum deviation observed for Arg75 (Fig. 4c,d).

The third binding hot-spot (pocket III), known to be the most critical of the three IKKβ/NEMO interaction sites, extends for approximately 16  Å^20^. IKKβ residues identified by Ala scanning mutagenesis to contribute significantly to binding include F734/L737/W739/W741/L742 and contact NEMO residues Glu89, Phe92, Leu93, Lys96, Phe97, Glu99, Ala100, L103 in chain B and Lys90′, Leu93′, Met94′, Phe97′, Ala100′, Arg101′, Val104′ in chain D. The two helix heptads that compose pocket III show a closing of the bun in the apo structure of 1.7–1.1 Å, and the largest rearrangement in side chain conformations (Fig. 4e,f). The helix axis in the apo structure, between Lys90′ and Val104′, rotates towards the center of the bundle by about 35 degrees. This clockwise shift about the coiled-coil axis relative to canonical is a known effect of the stutter discontinuity on the following heptads^34^. Consequently, several of the side chains now occupy the IKKβ binding pocket. The helix at Arg101′, which was disordered in the complex structure, is shifted toward the center to invade the pocket of W741 and L742; Phe97′ occupies the pocket of W739; Met 94′ closes in the site of F734 and L737. A large conformational change is observed in Phe97 in chain A, which formed the bottom of the hydrophobic pocket for W739 and now swings out to allow for the tighter packing of the two helices in the apo structure (the Cα-Cα distance for Phe97 is tighter by 2.9 Å, Supplementary Fig. S2). Leu93 now points to the F734 pocket in a closer *d*-*d’* packing and Lys96 swings towards the position of W739. Overall, looking down into pocket III the large hydrophobic cleft observed when IKKβ is bound (Fig. 4h) closes up, covered by the tighter helical packing and by polar surface residues of the coil (Fig. 4g).

The unbound NEMO structure was analyzed with CASTp^35^ to locate possible binding pockets on the topology of the protein structure. The most significant pocket identified by CASTp, with a surface area of 155  Å^2^, corresponds to pocket II, and comprises almost exactly the same residue side chains involved in the interaction with IKKβ, starting at residue Leu72 and extending to Gln83 (Supplementary Fig. S3). Because of the closing of the bun, only one other small pocket (surface area of 60  Å^2^) is identified, corresponding to the N-terminal portion of pocket III (residues 86–93).

### The I65M NEMO mutant mimics the open bun conformation

**Figure 5:**
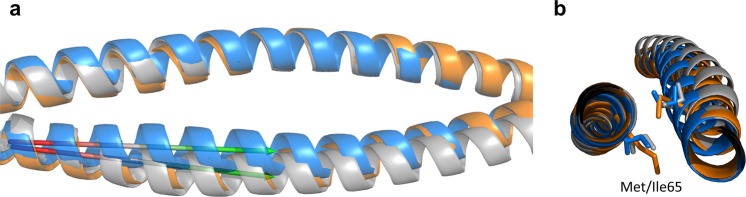
The I65M NEMO mutant mimics the open bun conformation. NEMO-EEAA (blue), NEMO-I65M (orange) and NEMO in the IKKβ complex (PDB: 3BRV, grey). (**a**) Proteins displayed as transparent ribbons encompassing residues 55–81 are superimposed on chain A. The angle between the helix axis for NEMO-EEAA and NEMO-I65M is shown by the arrows in the region 57–77: 5.66 degrees. (**b**) Effect of the Met/Ile mutation (shown as sticks).

The I65M mutation affects the opening of the hot-dog bun increasing the interhelical spacing of the three heptad repeats starting at residues 65, 72 and 79 to the values observed in the IKKβ-bound structure (Table 2). Figure 5a shows the hot-dog bun opening of NEMO-I65M in region 65–82 is similar to what observed in IKKβ-bound NEMO, and more open than in NEMO-EEAA. The longer side chain of Met65 extends deep in the interhelical cleft and the structure accommodates it by increasing the interhelical spacing by 1.4 Å and rotating the second Met65′ away from the center of the bun (Fig. 5b). This increased spacing seems to trigger the opening of the bun in the following heptads, till the end of the stutter “heptad” (79–82). The open region encompasses ligand binding pockets I and II. After residue 82, and approximately at the start of pocket III, the I65M structure closes back to resemble the apo structure, confirming the role of IKKβ in maintaining an open pocket III.

The conformation of ligand binding pockets I and II is similar in I65M and IKKβ-bound NEMO. The conformation of pocket III is instead almost identical in the I65M mutant and NEMO-EEAA structure, both in interhelical spacing and in side chain conformations, with an RMSD = 0.372 Å over all backbone atoms within residues 89–104. We observe the same movement of Phe97, with the aromatic rings swinging out to accommodate a tighter packing of the two helices.

NEMO-I65M and NEMO-EEAA crystallize in the same space group, with almost identical cell dimensions, but show some differences in crystal packing, which accommodate the different degrees of hot-dog bun opening. The region 63–81 of chain B, makes contacts with a symmetry molecule in NEMO-I65M which is shifted in register by a half helix-turn compared to the corresponding symmetry molecule in NEMO-EEAA, causing contacts with different symmetry amino acids.

In the GCN4 adaptors portion of the protein the NEMO-EEAA and NEMO-I65M have almost identical structures, with an RMSD of 1.272 Å over Cα carbons (residues 19–50 and 114–142), the most similar being the N-terminus (0.353 Å in 19–50). The structure of the GCN4 adaptor portion of NEMO-EEAA is also very similar to the structure of the native GCN4 leucine-zipper domain (PDB: 4DMD) with an RMSD of 0.595 Å in the N-terminal domain (NEMO-EEAA 24–51 and GCN4 3–30) and 1.223 Å in the C-terminal adaptor (NEMO-EEAA 114–141 and GCN4 5–32).

### Dynamic properties of the IKKβ-binding domain of NEMO

**Figure 6:**
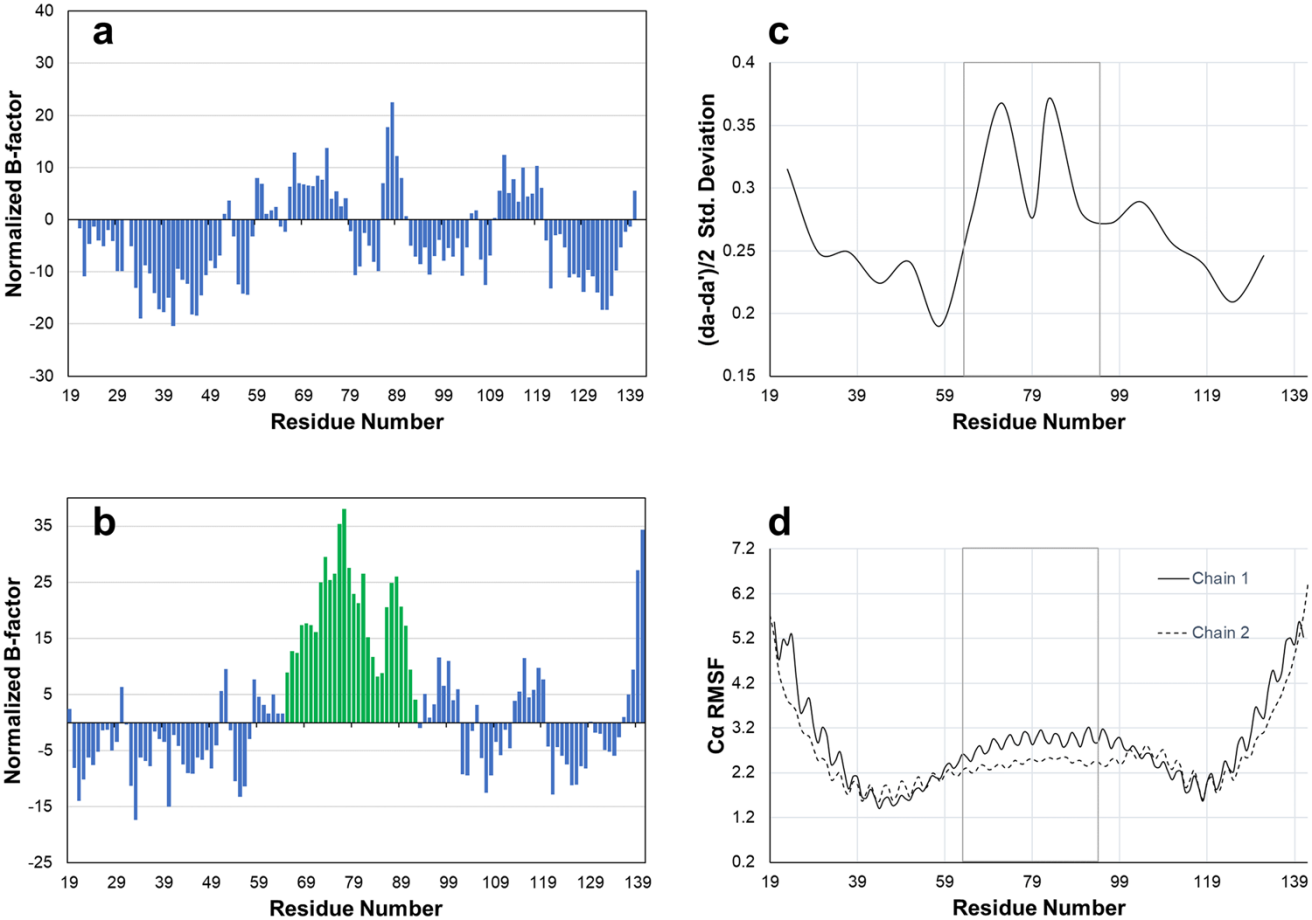
Analysis of NEMO dynamics. Left panels: normalized B-factors for chains A (**a**) and B (**b**) of the structure of NEMO-EEAA. The normalized B-factors are calculated from the average B-factor over all atoms for the residue, and then averaged over a moving window of three residues. Residues 65–92 of chain B are highlighted in green bars. Right panels: Analysis of the dynamics over the last 40 ns of the MD simulation of NEMO-EEAA. The region 65–92 is boxed. (**c**) Standard deviation of the average interhelical distances calculated as (*da* + *da’*)/2, using residue *d* in the center of each heptad as the abscissa value. (**d**) RMSF for Cα atoms.

We investigated if the central portion of the NEMO helix, an irregular coiled coil, displays local flexibility that facilitates binding to IKKβ. Analysis of normalized B-factors within the NEMO-EEAA structure shows increased values in residues 65–92 of chain B versus the remaining residues of the chain (all positive values, i.e. larger B-factor than average, Fig. 6a,b). Chain A of NEMO-EEAA does not display the same flexibility in region 65–92, with a distribution of positive and negative normalized B-factors. Molecular dynamics simulations of the NEMO-EEAA structure were run for 60 ns in a water box, starting from the solved crystal structure and including the coiled-coil adaptors, and analyzed for the last 40 ns, after equilibrium was reached. The trajectory analysis shows larger fluctuations in the interhelical spacing in NEMO’s central helix between residues 65–100 (Fig. 6c), encompassing the location of the three IKKβ-binding pockets. Similarly, the analysis of RMSF for Cα atoms indicates the expected flexibility at the protein termini, with rigid segments corresponding to the core of the coiled-coil adaptors (residues 37–50 and 114–122) and with increased mobility in the central NEMO helix approximately between residues 60–110 (Fig. 6d). Both helices in the dimer show similar flexibility, in agreement with experiencing a similar environment in the solvated state. The RMSFs also show a periodic fluctuation that reflects higher flexibility for coiled-coil solvent exposed residues in positions *b*, *c* and *f* (see Fig. 1).

## Discussion

Our structures of unbound NEMO reveal (i) how the NEMO N-terminal domain adopts a slightly underwound dimeric coiled-coil structure in absence of the ligand, with the central underwound portion hosting the three binding hot-spots, (ii) how the dimer transitions to a more open structure to accommodate the ligand and (iii) how the ligand binding region is intrinsically more flexible. We propose that the GCN4 adaptors stabilize the native structure of the NEMO IKKβ-binding domain similarly to what the full-length protein does, while providing a construct amenable to crystallization.

NEMO-EEAA and NEMO-I65M show a large improvement in folding, stability and IKKβ binding affinity compared to the wild type fragment of NEMO (44–111)^22^. Notably, a similar approach of enforcing the dimerization of NEMO (44–111) through oxidation at position 107 (L107C mutation) was reported to induce a stable α-helical coiled-coil structure that is preorganized to bind IKKβ with high affinity^36^. The results can be interpreted to indicate that the IKKβ binding domain of NEMO possesses an ordered structure in the unbound state when part of full length NEMO, but becomes conformationally heterogenous and unstable when truncated below residue 130.

### The designed coiled-coil adaptors retain the GCN4 structure and support a native-like structure in NEMO

The N-terminal coiled-coil adaptor folds into the expected ideal coiled-coil structure which is very similar to the native GCN4 structure (4DMD), confirming that the grafting and the heptad matching were successful. In addition, the ideal coiled-coil sequence propagates through the first two heptads of the proper NEMO sequence, resulting in a very stable N-terminal five heptads that contribute to the overall folding and stability of the NEMO construct. The stabilizing effect is reasonably similar or exceeds what provided by native NEMO residues 1–50 in the full protein^36^. The last three heptads of the adaptor region at the C-terminus fold in a regular coiled coil and are similar to the native GCN4 but with slightly larger RMSD values, possibly a cause of the discontinuities in the heptad repeats that propagate along the coiled coil. We suggest that the C-terminal coiled-coil adaptor mimics the native coiled-coil sequence predicted to induce NEMO dimerization in region 111–196^19^, within a much smaller footprint, more suitable for structural studies.

We also considered the effect that the coiled-coils adaptors have on the intervening NEMO structure, to confirm a native-like structure. NEMO regions outside of the binding pockets (Leu51-Ala64 and Val104-Leu109), maintain a very similar structure in the unbound form and when in complex with IKKβ (backbone and side chains conformations).

### The opening and closing of the NEMO dimer accommodate ligand binding

The most notable characteristic of the structure of NEMO (51–112) in the unbound form is a more compact packing of the two helices in the dimer, compared to the IKKβ bound structure, with an interhelical spacing reduced by up to 2.2 Å. As a result, the unbound NEMO structure buries much of the exposed surface area that constituted the interface to the bound ligand, and that would otherwise be unfavorably solvent exposed (Fig. 4e). Despite the tighter packing, the structure still represents an underwinding of the dimer supercoil, with larger interhelical spacing compared to an ideal coiled coil. The preference for a loser dimer is encoded in the heptad discontinuity in the NEMO sequence which presents a typical stutter (a four-residue insert) between residues Leu79-Phe82. Underwound coiled coils lack the knobs-into-holes packing in favor of non-close-packed cores, that are found in nature to terminate certain coiled-coil regions and to add flexibility to long coiled coils, as observed in myosin^34,37^. The effect of stutters, and other discontinuities, on protein structure and flexibility is thought to play a role both in protein assembly and in protein-protein interaction^34^, and seems in this case to facilitate the opening of the IKKβ binding pocket.

While the NEMO coiled coil underwinding in the apo structure encompasses the three binding hot-spots region, the region corresponding to the largest opening of any binding pocket, maximum interhelical distance increase upon ligand binding, and maximal flexibility is located at pocket II, coinciding with the stutter region. This may suggest that the open state of pocket II is a favored site for initial docking of the ligand, and that the substantial opening it undergoes upon ligand binding may trigger the opening of the adjacent binding pockets I and II. This is in agreement with a close to 1,000 fold decrease in binding affinity when a single hot-spot residue of pocket II is mutated: L719A mutant of IKKβ^20^.

### A hypothesis of mechanism for ligand binding

The hot-dog bun opening observed upon the conservative Ile/Met mutation^38^ can be interpreted as an inherent ability of the NEMO structure to easily transition between a closed and an open state. The NEMO-I65M construct is almost identical to the parent NEMO-EEAA and NEMO-CC constructs in helical and coiled-coil content, as well as in IKKβ binding affinity, but displays a lower thermal melting temperature (Fig. 2). The data suggest that NEMO-I65M unfolds more easily and more completely than the other two constructs, due to the prevention of a compact packing of the dimer at the beginning of the discontinuous region, caused by Met65, and destabilization of the packed regions of the coiled coil. A similar effect in thermal denaturation data was observed upon addition and removal of intermolecular disulfide bonds that respectively stabilize or destabilize the dimer in NEMO (44–111)^36^.

The same region that is wedged open by the I65M mutation is included in a region with increased B-factors within the unbound structure of NEMO-EEAA, extending from Ile65 to Leu92 and encompassing binding pockets I, II and the beginning of pocket III. The normalized B-factors of this region in chain B are significantly higher than in the remaining residues, and higher than the corresponding residues in chain A of NEMO-EEAA, indicating a higher flexibility of chain B and possibly a higher propensity to a conformational change to accommodate the ligand (Fig. 6). We measured the conformational change that each chain undergoes upon ligand binding in terms of RMSD. Chain A shows an RMSD of 0.909 Å (Cα atoms), between the apo and bound structure, while chain B undergoes a more significant change with an RMSD of 1.379 Å. The RMSD reflects a visible “bowing” of chain B upon ligand binding, to create a crescent shape and a larger opening to allow ligand binding, while chain A appears largely similar (Supplementary Video S1). The molecular dynamic simulations confirm that the NEMO region encompassing the three binding sites and characterized by a discontinuity in the coiled-coil sequence, is more dynamic than the ideal coiled coil in the adjacent adaptors.

The extensive opening of the NEMO dimer in the bound structure seems to be enforced by the length of IKKβ that encompasses the three binding hot-spots and largely accomplished by a conformational change in helix B. The data from the unbound structure suggests that the opening of the long cleft, or of the three binding pockets, occurs in a concerted manner, with the opening of one pocket affecting the opening of the neighboring ones. The data is consistent with the requirement of a long IKKβ fragment to retain high binding affinity^19^.

The three binding pockets do not appear to be preorganized in the closed hot-dog bun conformation of the NEMO unbound structure. At the major target site of the NBD peptide, pocket III, the hydrophobic groove which accommodates W739 and W741 is basically absent. The same is true for pocket I, although we know that the structure is prone to rearrange to an open form from both the complex structure and the NEMO-I65M structure. The only accessible pocket in the unbound conformation of NEMO is pocket II, which represents the point of maximum opening of the bun and where CASTp locates a pocket involving the same residues contacting the ligand in the bound form (Supplementary Fig. S3). The presence of this preformed pocket should be kept into consideration in the design and screening efforts of inhibitors of the IKKβ binding site on NEMO.

The primary target for the development of NEMO/IKKβ inhibitors has been up to now the NBD pocket (pocket III), mostly through the utilization of the NBD-peptide or small modifications of its native sequence^39,40^, or the design of NBD-peptide mimetics^9,41^. While the target of the NBD-peptide is reasonably expected to be pocket III, pocket II may play a role in binding small molecule inhibitors and still provide the desired inhibition of the NEMO/IKKβ interaction. Screening for small molecule inhibitors of the NEMO/IKKβ interaction has been reported, utilizing both in silico and *in vitro* systems^9,41–43^. The accessibility of a structure of NEMO in complex with the small molecule would at once verify that the desired binding pocket was in fact targeted and allow for the structure-based optimization of the initial inhibitor, a tool of fundamental importance when targeting a challenging protein-protein interaction site like NEMO^44,45^.

We developed a NEMO construct that, although incorporating only the IKKβ-binding domain of NEMO, provides stability and binding affinity similar to full length NEMO, while allowing for facile crystallization and structure determination. The NEMO unbound structure provides a new framework for targeting NEMO through structure-based approaches and holds high promise for the cocrystallization of NEMO in complex with small molecule inhibitors, to provide details on binding modes and allow further ligand improvements.

## Methods

### Construction of *E. Coli* expression vectors

The starting coiled-coil NEMO vector was obtained as described in^22^, NEMODcc. This vector was utilized for all subsequent vector’s construction. Mutations C76A, C95S, followed by E56,57 A and finally I65M were introduced by site-directed mutagenesis (QuikChange XL II mutagenesis kit) of NEMODcc, resulting in constructs NEMO-CC, NEMO-EEAA, and NEMO-I65M, respectively. The mutagenesis primers are listed in Supplementary Table S3.

The expression vector for IKKβ_KKRR_(701–745) was obtained as described in^22^.

### Expression and purification of NEMO constructs

The constructs were expressed in BL21 Star (DE3) *E*. *coli* cells. Cells were grown at 37 °C to OD_600_ = 0.8–1.0 before induction with 0.5 mM IPTG, and harvested after 6 hours. Cell pellets were resuspended in 25 mL of lysis buffer [20 mM Tris, 150 mM NaCl, 2 mM DTT, 1 mM PMSF, 1 μL Benzonase, pH 8.0]. A French press was used to lyse cells, and the lysate was denatured by adding urea to a concentration of 8 M. The clarified urea lysate was applied to a HisTrap 5 mL HP column (GE Healthcare) and washed using 20 mM Tris, 150 mM NaCl, 8 M urea, 2 mM DTT, pH 8.0. Urea was then removed using a wash buffer consisting of 20 mM Tris, 150 mM NaCl, 2 mM DTT, pH 8.0 and the target protein eluted with a 0–500 mM imidazole gradient. Bradford reagent was used to determine the protein concentration. His-tagged TEV protease was added at a ratio of 1 mg of TEV to 10 mg of His-tagged protein for TEV cleavage in dialysis buffer [20 mM Tris, 150 mM NaCl, 2 mM DTT, pH 8.0] overnight at 4 °C. The cleavage mixture was applied to the HisTrap column again to remove the His tag and TEV, followed by SEC with a Superdex75 16/60 column in 2 mM Tris, pH 8, 100 mM NaCl, and 2 mM DTT.

### Expression and purification of GST-NEMO(1–196)

The protein was produced and purified as described^22^. Briefly, cells were grown at 37 °C till induction and at 20 °C overnight. Clarified cell lysate was purified on a GSTrap 4B column (GE Healthcare), followed by SEC on a Superdex75 16/60 column (GE Healthcare).

### Expression, purification and labeling of IKKβ^KKRR^ (701–745)

The protein was expressed and purified in the same fashion as NEMO, with denaturing and on-column refolding followed by TEV cleavage and SEC. The protein was labeled with fluorescein isothiocyanate (FITC) as described^22^.

### Expression and purification of NEMO-I65M with SeMet labeling

The construct was expressed in B834 (DE3) *E*.* coli* cells, methionine auxotrophs. A 250 mL culture was grown at 37 °C to an OD_600_ of 3–4. Cells were centrifuged at 1,500 rpm for 10 minutes at 4 °C. Cells were then resuspended in M9 minimal media containing 3 g/L of ammonium chloride and 10 g/L dextrose. Cells were grown for 30 minutes before adding 100 µg/mL of selenomethionine, lysine, phenylalanine, and threonine as well as 50 µg/mL of leucine, isoleucine, and valine. Cells were grown for an additional 30 minutes before induction with 0.5 mM IPTG. Expression and purification followed the same protocol as the unlabeled constructs.

### Fluorescence Anisotropy

Direct binding assays were performed using N-terminally FITC-labeled IKKβ_KKRR_ (701–745). Measurements were obtained using a Tecan Infinity F500 plate reader at room temperature, and each experiment was run in triplicate. Each well contained 2 mM Tris, 100 mM NaCl, 2 mM DTT, pH 8.0, 0.1 mg/mL IgG and 0.5 mM Thesit, 30 nM FITC-labeled IKKβ_KKRR_ and NEMO proteins at varying concentrations. Plates were shaken for 20 seconds and incubated for 15 minutes at room temperature, prior to data collection. The anisotropy values, FA, were fitted by nonlinear least-squares regression to the modified quadratic binding equation shown below using MATLAB (R2017a, The MathWorks, Inc., Natick, Massachusetts, United States):

1 $${\rm{FA}}\,{\rm{observed}}={{\rm{FA}}}_{{\rm{\min }}}+{(\mathrm{FA}}_{{\rm{\max }}}\,-\,{{\rm{FA}}}_{{\rm{\min }}})\frac{{\rm{L}}+{{\rm{K}}}_{{\rm{D}}}+{\rm{P}}-\sqrt{{({\rm{L}}+{{\rm{K}}}_{{\rm{D}}}+{\rm{P}})}^{{\rm{2}}}-{\rm{4LP}}}}{{\rm{2L}}}$$

where FA_min_ is the anisotropy value of the reporter in absence of NEMO, FA_max_ is the maximum anisotropy value at saturating NEMO concentrations, L and P are the total concentrations of reporter and NEMO, and K_D_ is the dissociation constant.

### Circular Dichroism

Coiled-coil character and construct stability were assessed by circular dichroism (CD). All samples were analyzed at 10 µM protein concentration in 2 mM Tris, 100 mM NaCl, 2 mM DTT, pH 8.0. CD data was acquired over three accumulations from 198 to 250 nm at 20 °C. Melting curves were obtained through variable temperature scans at fixed wavelength (222 nm) from 20 °C to 90 °C at a 1 °C/minute ramp rate. The melting temperature T_m_ for each protein was estimated from the maximum of a plot of the first derivative of θ_222_ against temperature. Helical content was estimated from the CD spectra using K2D3^46^.

### Protein Crystallization of NEMO-EEAA and NEMO-I65M

Screening utilized MRC2 96 well sitting drop plates and a Formulatrix NT8 drop dispenser, with 200 nL drops at a 1:1 and 1:2 protein solution to mother liquor ratio. Fine screens were produced using a Formulatrix Formulator. Initial crystals were obtained with a PGA screen (Molecular Dimensions) using a protein concentration of 113 µg/ml in 2 mM Tris, 100 mM NaCl, 2 mM DTT, pH 8.0 and PGA-LM and varying PGA molecular weights and concentrations. A seed stock of NEMO-EEAA crystals was created from a fine screen condition of 0.1 M Tris pH 8.9, 6.5% PGA-LM, 3.6% PEG 20 K. Final crystals were obtained with seeding in 0.1 M Tris pH 8.0, 5.8% PGA-LM, 5.45% PEG 20 K. The crystals were cryoprotected by adding 10% 1,2 propanediol directly to the well prior to looping, and flash-frozen in liquid nitrogen. All crystal imaging used a Formulatrix Rock Imager 1000. Crystals of the NEMO-I65M mutant were obtained in identical conditions

### Data Collection and Structure Determination

Diffraction data were collected in cryogenic conditions at the AMX beamline at the National Synchrotron Light Source II (NSLS II), Brookhaven National Laboratory. The data were processed using XDS^47^.

The data for NEMO-EEAA is anisotropic, with data resolution varying in the b* and c* axis. We found that, although the uncorrected data could be phased by molecular replacement (MR-ROSETTA^48^), refinement of the structures stalled unless the data were anisotropically truncated. The data was initially elliptically truncated utilizing the Diffraction Anisotropy Server at UCLA^49^ and anisotropy was removed by anisotropic scaling, allowing phasing by molecular replacement using MRage^50^ within PHENIX^51^. The structure of the GCN4 leucine zipper domain in a dimeric oligomerization state (PDB: 4DMD)^52^ was used as a search model. For subsequent refinement the XDS processed data was submitted to the STARANISO^53^ server (http://staraniso.globalphasing.org/cgi-bin/staraniso.cgi) to define anisotropic diffraction cut-off surfaces without the assumption of an ellipsoid shape, and to apply an anisotropic correction factor to the amplitudes with Bayesian estimation^54^. The anisotropic diffraction cut-off was defined by a locally-averaged value of I/σ(I) > 1.2, as suggested by the server. The anisotropic truncation of the data, with the new limits of 1.885 Å, 2.101 Å and 2.548 Å along the a*, b* and c∗ axis respectively, resulted in an increase in the number of unique reflections to 19,560. The STARANISO output dataset was then merged with the original R_free_ flags, using Phenix’s Reflection File Editor. Manual and automated rebuilding and refinement utilized COOT^55^ and PHENIX.

The data for NEMO-I65M was phased by molecular replacement, using a preliminary structure of NEMO-EEAA as a search model. Manual and automated rebuilding and refinement utilized COOT and PHENIX. To check for possible effects of model bias, composite omit maps were calculated with PHENIX, excluding 10% of the data. Data collection statistics and refinement parameters are given in Table 1.

Normalized B-factors were calculated as^56–58^:

2 $$B{N}^{\text{'}}=\frac{B-{B}_{ave}}{\frac{{B}_{std}}{\sqrt{n}}\sqrt{\frac{N-n}{N-1}}}$$

where *B* is the B-factor per residue (calculated as the average of the single atom B-factors), *B*_*ave*_ and *B*_*std*_ were computed on all residues of the structure (*N*), and *n* is the number of residues of the examined helix (chain A or chain B). Normalized B-factors reported in Fig. 6 are averaged over a moving window of three residues.

The figures were generated using PyMOL^59^. Surface rendering displays the solvent-accessible surface area with a probe radius of 1.4 Å. Coloring by hydrophobicity uses the Eisenberg hydrophobicity scale^60^. The video was generated with the UCSF Chimera package^61^ with the Morph Conformations function. Two molecular models of the NEMO (51–110) sequence adopting the conformation observed in NEMO-EEAA and NEMO-I65M were calculated using SWISS-MODEL^62^ and NEMO-EEAA and NEMO-I65M as templates. NEMO(51–110) is shown morphing between the three conformations: NEMO-EEAA, NEMO-I65M and 3BRV.

### MD Simulation

All-atom molecular dynamics simulations of NEMO-EEAA were performed with NAMD, version 2.10b, release 2014^63^, with the CHARMM27 force field^64^. The initial protein model is the crystal structure of NEMO-EEAA including the coiled-coil adaptor regions, which was placed in a cubic box with a 1.5 nm edge distance. The protein was solvated with 16053 water molecules utilizing the TIP4 water model^65^. The starting hydrated model was relaxed to address steric clashes and inappropriate geometry with steepest descent energy minimization. The system was equilibrated for 100 ps of MD simulation at 300 K with a time step of 2 fs and a pressure of 1 bar. The MD simulation was run for 60 ns, utilizing the LINCS algorithm^66^ to constrain all bond lengths and the Particle Mesh Ewald method^67^ to calculate long-range electrostatic interactions. For temperature and pressure control, the Berendsen and Parrinello-Rahman coupling algorithms^68,69^ were used, respectively. The last 40 ns of the simulation were utilized for analysis, to allow the system to come to equilibrium (as determined by analysis of the RMSD along the trajectory). Analysis utilized custom Tcl scripts. Interhelical spacing was calculated as *ad* distances, as in Fig. 1.

### Quantification and Statistical Analysis

First derivatives for the determination of T_m_ from CD thermal melting curves were calculated using Microsoft Excel. Data for the K_D_ determination were fitted using MATLAB, errors are reported as 95% confidence bounds. The B-factors averages and standard deviations in Fig. 6 were calculated using Microsoft Excel. The *ad* interatomic distances were obtained from the MD simulation trajectories using custom Tcl scripts.

## Supplementary information

Supplementary Information

Simulation of NEMO conformational change upon ligand binding

## Data Availability

Protein Data Bank: The atomic coordinates and structure factors for the crystal structures of NEMO-EEAA and NEMO-I65M are deposited under accession codes 6MI3 and 6MI4, respectively. Additional data are available upon request from the corresponding author (M.P.).
