# Supplementary material for: The IKK-binding domain of NEMO is an irregular coiled coil with a dynamic binding interface
Source: Sci Rep. 2019 Feb 27;9:2950. doi: 10.1038/s41598-019-39588-2 (PMC6393490; doi:10.1038/s41598-019-39588-2)
Supplement: Supplementary file 1 — Supplementary Information [file 41598_2019_39588_MOESM1_ESM.pdf]

Supplementary Information

The IKK-binding domain of NEMO is an irregular coiled coil with a dynamic binding interface

Adam H. Barczewski, Michael J. Ragusa, Dale F. Mierke and Maria Pellegrini*

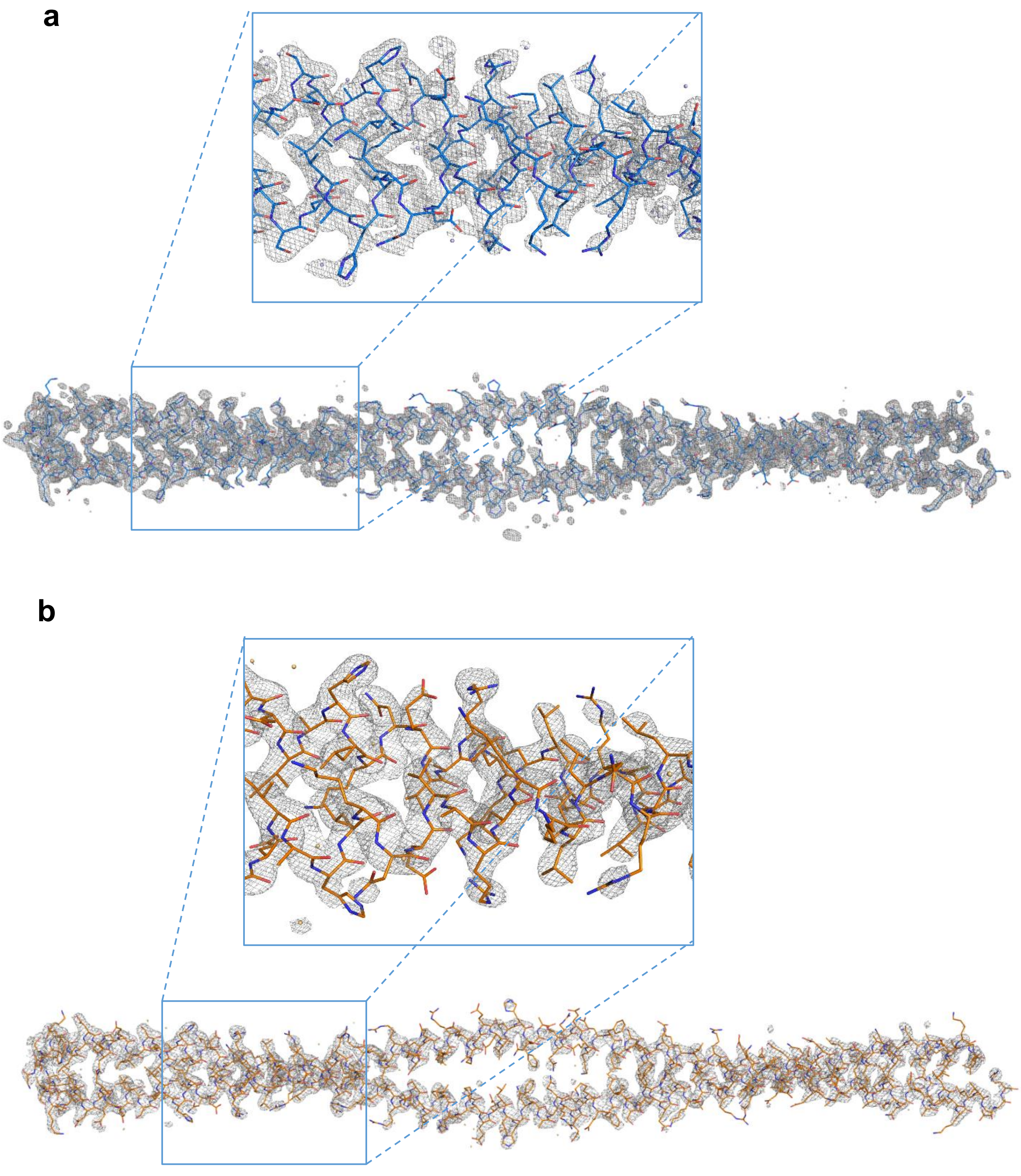

Supplementary Figure S1. Composite omit maps of NEMO-EEAA and NEMO-I65M**.** Models of NEMO, shown as sticks, overlaid to the composite omit map 2F_o-F_c, calculated with PHENIX omitting 10% of the atoms at a time, contoured at 1σ. (**a**) NEMO-EEAA in blue. (**b**) NEMO-I65M in orange. A zoom region is indicated.

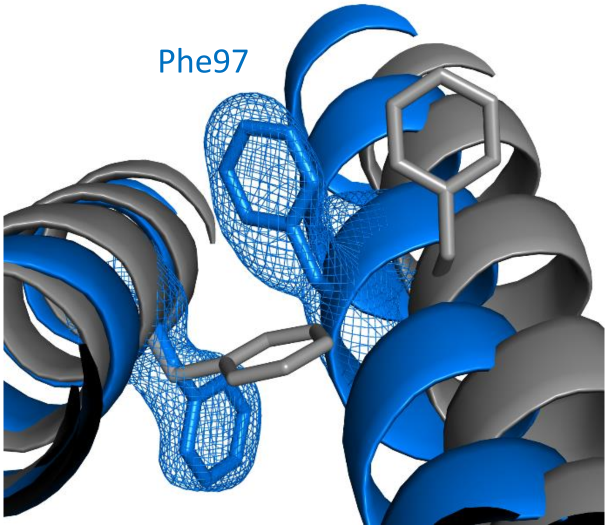

Supplementary Figure S2. Conformational change of Phe97. NEMO-EEAA in blue, overlaid to NEMO in the IKKβ complex (PDB: 3BRV) in grey, using chains A of NEMO-EEAA and B of 3BRV. The conformational change experienced by Phe97 is highlighted. The electron density map for Phe97 is contoured at 1σ.

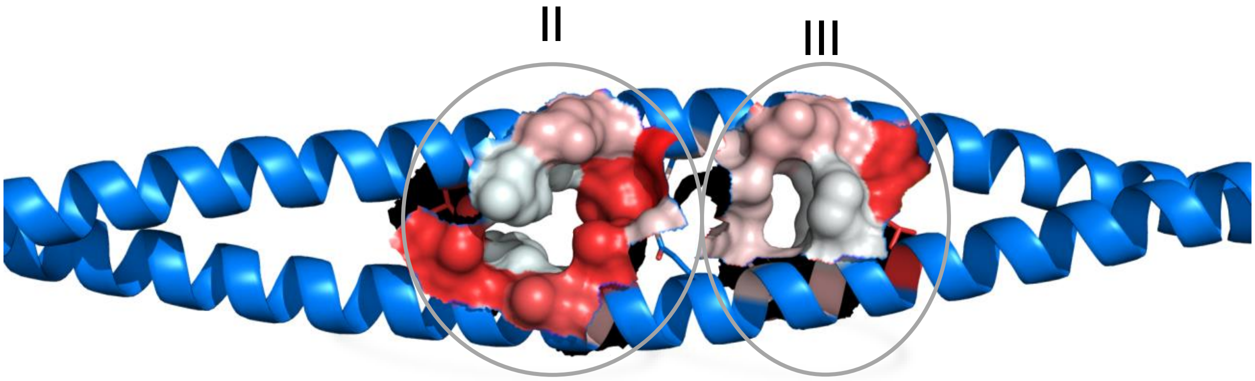

Supplementary Figure S3. Binding pockets identified by CASTp on NEMO-EEAA. Pockets are labeled as II and III as in the text. The pocket surface is colored by hydrophobicity (red = hydrophobic, white = polar). NEMO is displayed as a blue ribbon in the region 52-108.

Supplementary Table S1 Sequences of the NEMO constructs. In italic the coiled-coil adaptors sequences. Mutations are underlined.

Construct	Sequence
**NEMO(51-112)**:<br>wild type	LQRCLEENQELRDAIRQSNQILRERCEELLHFQASQREEKEFLMCKFQEARKLV<br>ERLGLEKL
**NEMO-CC**:<br>double coiled-coil,<br>C76A, C95S<br>mutant	*GSWSVKELEDKNEELLSEIAHLKNEVARLKKL*LQRCLEENQELRDAIRQSNQIL<br>RERAEELLHFQASQREEKEFLMSKFQEARKLVERLGLEKL*ELEDKNEELLSEIA*<br>*HLKNEVARLKKLVGER*
**NEMO-EEAA**:<br>double coiled-coil,<br>C76A, C95S,<br>E56A, E57A mutant	*GSWSVKELEDKNEELLSEIAHLKNEVARLKKL*LQRCLAANQELRDAIRQSNQIL<br>RERAEELLHFQASQREEKEFLMSKFQEARKLVERLGLEKL*ELEDKNEELLSEIA*<br>*HLKNEVARLKKLVGER*
**NEMO- I65M**:<br>double coiled-coil,<br>C76A, C95S,<br>E56A, E57A, I65M<br>mutant	*GSWSVKELEDKNEELLSEIAHLKNEVARLKKL*LQRCLAANQELRDAMRQSNQIL<br>RERAEELLHFQASQREEKEFLMSKFQEARKLVERLGLEKL*ELEDKNEELLSEIA*<br>*HLKNEVARLKKLVGER*

Supplementary Table S2 Coiled-coil prediction by PairCoils2

**Position**	**Residue**	**Register**	**P-score**
19	G	d	0.01103
20	S	e	0.00368
21	W	f	0.00353
22	S	g	0.00091
23	V	a	0.0005
24	K	b	0.0005
25	E	c	0.0005
26	L	d	0.0005
27	E	e	0.0005
28	D	f	0.0005
29	K	g	0.0005
30	N	a	0.0005
31	E	b	0.0005
32	E	c	0.0005
33	L	d	0.0005
34	L	e	0.0005
35	S	f	0.0005
36	E	g	0.0005
37	I	a	0.0005
38	A	b	0.0005
39	H	c	0.0005
40	L	d	0.0005
41	K	e	0.0005

42	N	f	0.0005
43	E	g	0.0005
44	V	a	0.0005
45	A	b	0.0005
46	R	c	0.0005
47	L	d	0.0005
48	K	e	0.0005
49	K	f	0.0005
50	L	g	0.0005
51	L	a	0.0005
52	Q	b	0.0005
53	R	c	0.00054
54	C	d	0.00103
55	L	e	0.00181
56	A	f	0.00198
57	A	g	0.00198
58	N	a	0.00198
59	Q	b	0.00198
60	E	c	0.00198
61	L	d	0.00198
62	R	e	0.00243
63	D	f	0.00243
64	A	g	0.00243
65	I	a	0.00243
66	R	b	0.0036
67	Q	c	0.00383
68	S	d	0.00469
69	N	e	0.00469
70	Q	f	0.00637
71	I	g	0.01096
72	L	a	0.01096
73	R	b	0.01673
74	E	c	0.01673
75	R	a	0.0264
76	A	b	0.02751
77	E	c	0.02751
78	E	d	0.03464
79	L	e	0.03974
80	L	b	0.04907
81	H	f	0.04967
82	F	g	0.04967
83	Q	a	0.04967
84	A	b	0.03631
85	S	c	0.0245

86	Q	d	0.02033
87	R	e	0.01743
88	E	f	0.01713
89	E	g	0.01713
90	K	a	0.01713
91	E	b	0.0153
92	F	c	0.01356
93	L	d	0.00886
94	M	e	0.00886
95	S	f	0.00667
96	K	g	0.0057
97	F	a	0.0057
98	Q	b	0.0047
99	E	c	0.00347
100	A	d	0.00347
101	R	e	0.00347
102	K	f	0.00337
103	L	g	0.00337
104	V	a	0.0024
105	E	b	0.0024
106	R	c	0.00231
107	L	d	0.00222
108	G	e	0.00222
109	L	f	0.00176
110	E	g	0.001
111	K	a	0.001
112	L	b	0.001
113	E	c	0.001
114	L	d	0.001
115	E	e	0.001
116	D	f	0.001
117	K	g	0.001
118	N	a	0.001
119	E	b	0.001
120	E	c	0.001
121	L	d	0.001
122	L	e	0.001
123	S	f	0.001
124	E	g	0.001
125	I	a	0.001
126	A	b	0.001
127	H	c	0.001
128	L	d	0.001
129	K	e	0.001

130	N	f	0.001
131	E	g	0.001
132	V	a	0.001
133	A	b	0.001
134	R	c	0.001
135	L	d	0.001
136	K	e	0.001
137	K	f	0.001
138	L	g	0.001
139	V	a	0.001
140	G	b	0.00237
141	E	c	0.00237
142	R	d	0.00288

Supplementary Table S3 Mutagenesis primers

C76A Primers	5' - CAG ATT CTG CGG GAG CGC GCC GAG GAG CTT CTG CAT TTC - 3′<br>3' - GAA ATG CAG AAG CTC CTC GGC GCG CTC CCG CAG AAT CTG - 5′
C95S Primers	5' - GAG AAG GAG TTC CTC ATG AGC AAG TTC CAG GAG GCC AGG -3′<br>3' - CCT GGC CTC CTG GAA CTT GCT CAT GAG GAA CTC CTT CTC -5′
E56A E57A Primers	5′ - CTC CAG CGC TGC CTG GCG GCG AAT CAA GAG CTC CGA GAT GCC - 3′<br>3′ - GGC ATC TCG GAG CTC TTG ATT CGC CGC CAG GCA GCG CTG GAG - 5′
I65M Primers	5′ - GCG AAT CAA GAG CTC CGA GAT GCC ATG CGG CAG AGC AAC CAG ATT CTG CGG - 3′<br>3′ - CCG CAG AAT CTG GTT GCT CTG CCG CAT GGC ATC TCG GAG CTC TTG ATT CGC - 5′
